# Graphene Quantum Dot-Added Thin-Film Composite Membrane with Advanced Nanofibrous Support for Forward Osmosis

**DOI:** 10.3390/nano12234154

**Published:** 2022-11-24

**Authors:** Haleema Saleem, Pei Sean Goh, Asif Saud, Mohammad Aquib Wakeel Khan, Nazmin Munira, Ahmad Fauzi Ismail, Syed Javaid Zaidi

**Affiliations:** 1 UNESCO Chair on Desalination and Water Treatment, Center for Advanced Materials (CAM), Qatar University, Doha P.O. Box 2713, Qatar; 2 Advanced Membrane Technology Research Centre, School of Chemical and Energy Engineering, Universiti Teknologi Malaysia, Johor Bahru 81310, Malaysia

**Keywords:** banyan tree leaves, graphene quantum dots, solution blow-spinning, forward osmosis, nanofiber membranes

## Abstract

**Highlights:**

**What are the main findings?**

- The current study establishes that banyan tree leaf-derived B-GQD-incorporated thin-film composite membranes with a SBS NFM substrate show an enhanced FO performance regarding water flux, salt rejection, and chlorine re-sistance ability.

**What is the implication of the main finding?**

- The membranes developed in the current study have great prospective applications in wastewater treatment, water purification, and desalination.
- GQD synthesis from banyan tree leaves was conducted by employing a simple hydrothermal technique.
- No organic solvent or reducing agent was utilized in this GQD synthesis.
- The GQDs were incorporated in a TFC membrane for the FO process.
- The TFC membrane’s support was fabricated by the SBS process.
- The 0.050-B-GQD/PA TFNC membrane demonstrated excellent FO performance and chlorine resistance.

**Abstract:**

Forward osmosis (FO) technology for desalination has been extensively studied due to its immense benefits over conventionally used reverse osmosis. However, there are some challenges in this process such as a high reverse solute flux (RSF), low water flux, and poor chlorine resistance that must be properly addressed. These challenges in the FO process can be resolved through proper membrane design. This study describes the fabrication of thin-film composite (TFC) membranes with polyethersulfone solution blown-spun (SBS) nanofiber support and an incorporated selective layer of graphene quantum dots (GQDs). This is the first study to sustainably develop GQDs from banyan tree leaves for water treatment and to examine the chlorine resistance of a TFC FO membrane with SBS nanofiber support. Successful GQD formation was confirmed with different characterizations. The performance of the GQD-TFC-FO membrane was studied in terms of flux, long-term stability, and chlorine resistance. It was observed that the membrane with 0.05 wt.% of B-GQDs exhibited increased surface smoothness, hydrophilicity, water flux, salt rejection, and chlorine resistance, along with a low RSF and reduced solute flux compared with that of neat TFC membranes. The improvement can be attributed to the presence of GQDs in the polyamide layer and the utilization of SBS nanofibrous support in the TFC membrane. A simulation study was also carried out to validate the experimental data. The developed membrane has great potential in desalination and water treatment applications.

## 1. Introduction

Over the last decade, freshwater requirements have extremely intensified around the globe due to the increasing growth in human population, rapid industrialization and urbanization, and enhanced risk from climatic variations [1,2,3,4]. Desalination and the treatment of wastewater are considered to be the two major prospective solutions to the challenges related to the freshwater shortage [5,6,7,8,9]. Forward osmosis (FO) is an advanced membrane-based separation process that has received increasing interest for various applications in the last several years [10,11]. The FO process is driven by the osmotic pressure difference between a diluted feed solution (FS) and a concentrated draw solution (DS) across a semipermeable membrane [12,13,14,15,16]. Currently, FO technology has been used in different areas such as desalination, water treatment, power generation, and food processing [17,18]. A standard thin-film composite (TFC) FO membrane possesses a porous membrane support layer that enables the appropriate transport of water and a thin selective polyamide (PA) layer accountable for salt rejection [19,20]. Regardless of the high potential demonstrated by the FO process, numerous challenges must be overcome to promote the commercial application of FO technology [21,22]. Some of the challenges commonly experienced in FO processes are a high reverse solute flux (RSF), low water flux, internal concentration polarization, poor mechanical properties of membranes, severe energy utilization for DS regeneration, and water recovery from a DS [23,24,25]. Additionally, the most frequently used membrane in FO applications, i.e., the PA thin-film composite (TFC) membrane, is extremely sensitive to chlorine [26,27]. These problems can be mitigated through innovative membrane design. In recent times, nanomaterials have been successfully applied in various sectors for environmental remediation [28,29]. Numerous modification methods, such as nanomaterial inclusion into a selective layer or membrane substrate and membrane surface coating, have been examined to enhance the PA TFC membrane performance for specific applications [30,31,32,33]. Nanomaterials can be included into a selective PA layer by adding them into monomer solutions to develop thin-film nanocomposite (TFNC) membranes [34,35]. Several research works have evidenced the enhanced membrane characteristics of TFNCs in terms of their water permeability, solute selectivity, chlorine resistance, fouling resistance, mechanical stability, and chemical stability in numerous membrane-based separation processes [36,37,38,39,40].

The application of nanofibers for membrane fabrication has received rising interest in the past decade. This is due to their high porosity, superior stability, and excellent chemical resistance [41]. Several previous studies on the application of nanofiber membranes (NFMs) in separation processes such as RO [42], FO [43], nanofiltration [44], ultrafiltration [45], and membrane distillation [46] have been reported. To overcome the current issues related to the membranes used in the FO process and to further improve the performance of the membranes in the FO process, i.e., enhancing the water flux and decreasing the RSF, the NFM support of superior porosity is recommended [47]. Despite the fact that electrospinning is the conventional nanofiber production method [43], the nanofibers prepared via this method demonstrate a low tortuosity due to their interconnected void spaces [47]. Among the various polymers used for nanofiber preparation, PES has received increased research attention owing to its good environmental resistance, outstanding mechanical properties, thermo-oxidative stability, good hydrolytic stability, good biocompatibility, non-biodegradability, and thermal stability [48,49]. Although the electrospinning technique is appropriate for the industrial production of nanofibers, its decreased rate of fiber production is considered a major disadvantage [41]. Recently, an advanced nanofiber production technique, i.e., solution blow-spinning (SBS), was proposed; this technique merges the features of electrospinning and melt-blowing techniques, and it has been established as an alternative for the fabrication of fibers. The SBS technique has a short preparation time, high yield, and comparatively more polymers and spinning solutions can be produced in relation to other techniques. Furthermore, the SBS system is exceptionally easy, and the diameter of nanofibers achieved using the SBS process is similar to the diameter of nanofibers prepared using the electrospinning process [41].

There have a significant number of research studies performed in the field of nanoparticles, especially regarding the carbon-based ones in the FO process, and this has been proven by the increasing number of studies that reported every year on the FO subject [39,50,51]. Recently, graphene quantum dots (GQDs) have been increasingly used as nanofillers in the field of membrane-based technology owing to their simple and low-cost preparation and their superior physicochemical properties such as excellent antifouling properties, good biocompatibility, high chemical stability, and small particle size [52,53]. GQDs are quasi-spherical, zero-dimensional nanoparticles with particle sizes ranging from 2 to 20 nm and exceptional hydrophilic properties because of the availability of a significant number of epoxy, hydroxyl, and carboxyl groups on the GQD surfaces [54]. The surface functional groups in GQDs promote water solubility and support additional surface functionalization. These attractive features of GQDs offer opportunities to enhance the membranes used for nanofiltration (NF) [52], RO [55], and FO [56,57]. A few studies have reported on the application of commercial GQDs and GQDs developed from chemicals for FO applications [56,57,58]. Xu et al. [57] proved that the separation ability of an FO TFC membrane increased upon the addition of graphene quantum dots, prepared from citric acid, into the selective polyamide layer. Furthermore, it was found that the inclusion of GQDs into the membranes could enhance the water permeability of the membranes by almost 50% to 200% [59,60]. Idrees et al. [61] modified a commercial PA membrane by grafting nitrogen-doped GQDs to improve its water flux, chlorine resistance, and salt rejection relative to the TFC membrane. Although different naturally occurring carbon species such as carbon black [62,63], candle soot [64], carbon fibers [65], and coal [66] can be used as a carbon precursor to develop GQDs, these carbon precursors are completely related to non-renewable resources and may not be sufficiently available in the forthcoming centuries. These limitations have stimulated the synthesis of GQDs from extremely sustainable, very renewable, natural, and low-cost sources such as green plants, which are the basis of most of the globe’s ecologies [67,68,69,70]. In a previous study, we developed GQDs from Eucalyptus tree leaves, but we obtained a lesser yield. Hence, in this study, we selected a new precursor to obtain a higher GQD yield. The *Ficus benghalensis* (banyan tree), the national tree of India, belongs to the family of *Moraceae* and the genus of *Ficus*. There are more than 800 species and around 2000 varieties of the *Ficus* species available [71], and in recent times, it has been observed that nanomaterials such as ZnO [72], CuO [72], ZrO_2_ [71], and CNTs [73] can be obtained from the leaves of the banyan tree. Banyan tree extracts are primarily composed of hydrocarbons comprising low amounts of oxygen [74], so they can serve as good precursors for preparing GQDs.

In the current study, GQDs synthesized from banyan tree leaves were incorporated into the PA layer of thin-film composite FO membranes. To the best of our knowledge, the current study represents the first effort of preparing GQDs from banyan tree leaves, which is a very environmentally friendly procedure that does not require the usage of any chemicals during its synthesis. The FO performance of the GQD-incorporated TFC membrane with PES-based NFM support fabricated via the SBS technique was evaluated in the present work. Furthermore, the chlorine resistance ability of the GQD-incorporated nanocomposite membrane after exposing the membrane to a sodium hypochlorite solution was analyzed. This was the first attempt to evaluate the chlorine resistance of TFC/TFNC FO membranes with SBS NFM support. Moreover, a simulation study was also carried out to validate the experimental data.

## 2. Methods and Materials

### 2.1. Materials

The N-methyl-2pyrrolidone (NMP), polyethersulfone (PES), and toluene employed to prepare the nanofibers were purchased from Sigma Aldrich, St. Louis, MO, USA. Metaphenylenediamine (MPD) (>99%), trimesoyl chloride (TMC) (>98%), n-hexane, and deionized (DI) water for the interfacial polymerization (IP) process were obtained from Merck, Kenilworth, NJ, USA. Banyan tree leaves obtained from the campus of Qatar University, Doha, Qatar were used for GQD preparation. Furthermore, the sodium chloride (NaCl) (99.50%) employed in the forward osmosis experiments and sodium hypochlorite (5–6% concentrated solution) for chlorine resistance analysis were purchased from Research Lab India, Islampur, India.

### 2.2. GQD Synthesis from Banyan Tree Leaves

**Figure 1:**
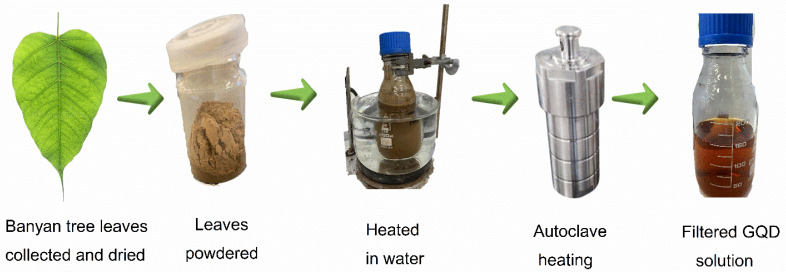
The stages involved in the synthesis of GQD from banyan tree leaves.

GQDs were synthesized from banyan tree leaves by adopting the preparation method reported by Saleem et al. [30]. The powder of banyan tree leaves (5.0 g) was acquired via the ball-milling of leaves, and this powder was heated in DI water (200 mL) for 1 h at 80 °C temperature. Then, this solution underwent centrifugation at 10,000× *g* of relative centrifuge force (RCF) for 10 min to separate the solid remains. The topmost liquid collected after the centrifugation was subsequently filtered to remove any additional existing solids. Subsequently, the stirring and sonication of the solution were performed for thirty minutes at a 25 °C temperature. This solution was then kept in an autoclave at 200 °C for thirteen hours. After this hydrothermal treatment, a black precipitate was formed in the brownish suspension. The black precipitate was separated, and the remaining suspension underwent centrifugation at an RCF of 10,000× *g* for 20 min. Subsequently, the supernatant was separated, and subsequently subjected to washing several times. The solution was consequently well-filtered and then appropriately dried at 75 °C. The GQDs synthesized from the banyan tree leaves were termed ‘B-GQDs’. A diagrammatic representation of the synthesis stages of B-GQD is shown in Figure 1. The GQD yield we obtained for the preparation from Eucalyptus tree leaves was 38.4% [30], whereas the GQD yield we obtained for the preparation from banyan tree leaves was 49.2%. Thus, it can be confirmed that banyan tree leaves is a better precursor than Eucalyptus tree leaves and neem leaves (25.2% yield) [67].

### 2.3. PES-Based Nanofiber Fabrication via SBS Technique

**Figure 2:**
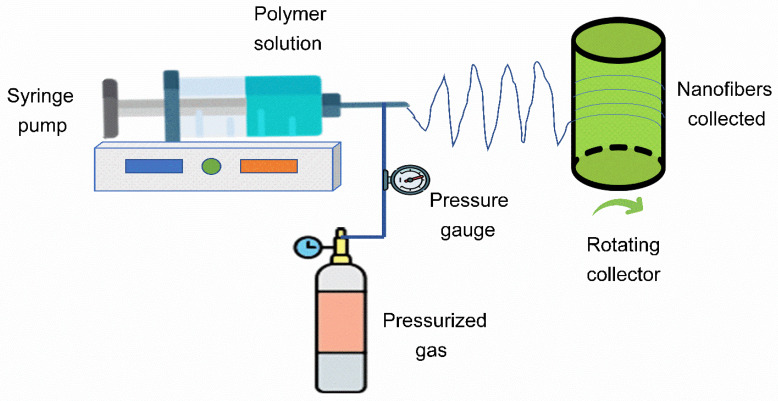
Schematic representation of the SBS system.

The SBS technique was used to prepare a PES-based nanofiber membrane as schematically shown in Figure 2. Firstly, 25 wt.% of PES pellets were added to a 2:1 wt.% solvent mixture of NMP/toluene and mixed properly to obtain a polyethersulfone solution. The polymer solution feeding rate of the SBS system was kept at 8 mL/h, with an air pressure of 2 bars and a voltage of 20 kV. The polymer solution was subsequently pumped through a gauge needle and placed inside a concentric nozzle at an operating distance of 50 cm. Across the concentric exterior nozzle, a pressurized velocity gas was allowed to pass through the interior nozzle through which the polymeric solution was pumped. To obtain uniform nanofiber distribution and deposition, a deposition time of 10 min was chosen. As the polymer solution approached the nozzle tip, it was extended due to the shear effect created by compressed air, hence forming a nanofibrous layer.

### 2.4. Heat-Press Post-Treatment for PES-Based Nanofiber Mat

The PES-based NFM was heat-pressed in post-treatment to enhance its morphological and mechanical properties such as robustness and resistance to wetting [45]. This type of heat treatment could improve the water permeate flux of the NFM in desalination and water purification applications, and, as a result of the nanofiber compaction, the contact angle is decreased [75]. The nanofiber mat had thickness of almost 700–800 microns. The heat-press treatment process functions based on the hardening principle, enhancing the mechanical characteristics of the polymer by subjecting it to a temperature above or its glass transition temperature. The PES-based NFM was supported using a polyester backing layer, and this NMF was positioned between a pair of steel frames. The prepared nanofiber mats were subjected to a heat-press post-treatment for 10 min at 150 °C under a load of 1.0 tons/m^2^. Then, the steel frames were separated from the machine and allowed to cool.

### 2.5. Membrane Fabrication (TFC and TFNC)

**Figure 3:**
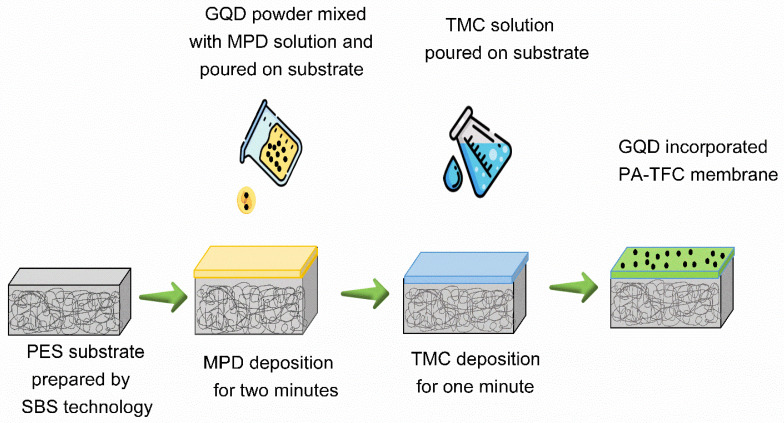
Diagrammatic representation of TFNC membrane preparation.

For the preparation of pristine TFC membranes, the PA layer was developed with an interfacial polymerization process on the surface of the NFM substrate, as presented in Figure 3. Initially, the NFM was stored in DI water for about 12 h before performing the IP process. For the IP process, a 2.0 wt.% MPD/DI water solution was transferred onto the NFM surface and retained for 2 min; subsequently, the extra MDP solution was rolled out from the surface of the NFM support by employing a rubber roller. Then, a 0.15 wt.% TMC/n-hexane solution was added to the surface of the membrane for 1 min. Subsequently, the TFC membrane was dried in air for 2 min, dried in an oven for 3 min at 90 °C, and then stored in DI water prior to FO performance testing.

To prepare the TFNC membranes, three different loadings of B-GQDs, i.e., 0.025, 0.050, and 0.075 wt.%, were first added to the MPD solution. The procedure for the fabrication of TFNC membranes was the same as that of the TFC membrane. The TFNC membranes were named 0.025-B-GQD/PA TFNC, 0.050-B-GQD/PA TFNC, and 0.075-B-GQD/PA TFNC for B-GQD loadings of 0.025, 0.050, and 0.075 wt.%, respectively.

### 2.6. Characterization of B-GQDs and the TFC/TFNC Membranes Developed

The characterization of B-GQDs was carried out to prove their effective preparation. Moreover, characterizations were performed on the TFC and TFNC membranes to examine the impacts of B-GQD inclusion into the PA layer of the membrane.

UV–vis absorption spectrophotometry and photoluminescence (PL) analysis were carried out to confirm the optical characteristics of the developed B-GQDs. The UV–vis absorption spectrophotometer used in this study was a UV Biochrom Spectrophotometer. The PL characteristics of the B-GQDs were examined using a FluoroMax-4 Spectrofluorometer from Horiba. GQDs exhibit tunable PL behavior through the manipulation of edge functionality under various synthesis conditions. The employed Fourier transform infrared (FTIR) instrument was a 760 Nicolet, and it identified the different organic and inorganic groups in the B-GQDs. Furthermore, transmission electron microscopy (TEM) (FEI Tecnai G2 S-Twin 200 kV FEG instrument) was used to confirm the morphology of the developed B-GQDs. The powder B-GQD samples were dispersed in isopropanol for 10–20 min using a bath sonicator, and 1–2 drops of the solution were drop-cast onto the carbon film a 200 copper mesh and kept for drying, followed by TEM analysis using an FEI Tecnai G2 S-Twin 200 kV FEG instrument. Furthermore, a Thermo Fisher Scientific DXR Microscope was employed to perform the Raman spectroscopy analysis of the BGQD samples.

For the characterization of TFC and TFNC membranes, the scanning electron microscopy (SEM) instrument employed in the current study was a Nova Nano SEM 450. The membrane surface characterization was carried out in SEM. An IV 3100 SPM Veeco Metrology Nasoscope was used to examine the surface roughness of the TFC and TFNC membranes. Moreover, the development of the polyamide layer and the proper inclusion of B-GQDs in the membranes were examined using FTIR (760 Nicolet). An OCA 708381-T, LMS Scientific instrument was used to examine the water contact angle of the prepared membranes.

### 2.7. Operation of FO System

**Figure 4:**
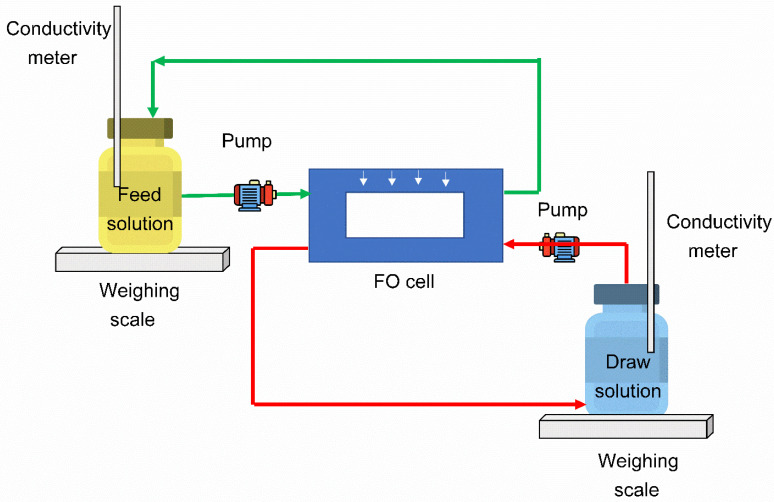
Schematic representation of the FO system.

In this study, we used a Sterlitech Cross flow CF042F cell, along with two flow meters, to monitor the FS flow and DS flow (Figure 4). In this FO unit, the rate of variation of mass and conductivity change with time was used to examine the FO performance.

In the current research work, 1.5 M of sodium chloride in 1 L of DI water was employed as a draw solution and 0.1 M of sodium chloride in 3 L of DI water was employed as a feed solution. The Sterlitech cell CF042 was employed, and the membrane samples were cut according to cell size. Initially, the precut membranes were properly preconditioned in deionized water for at least 10 h and then washed using distilled water prior to being kept within the Sterlitech cell. All forward osmosis tests were carried out in the AL-DS and AL-FS membrane orientations. During the start of each FO test, the feed solution and draw solution sides were backwashed using 2 L of deionized water on both sides. A 30 min backwashing process was performed to clean the membrane and the system. Subsequently, all FO experiments were carried out using 0.1 M of NaCl as the FS and 1.5 M of sodium chloride as the DS. The temperature of the feed and draw solutions was maintained at a constant of 25 °C throughout the FO tests. DI water backwashing was carried out after each FO test to control scale formation and fouling in the FO system. In all FO tests, membranes with different loadings of B-GQDs were tested with all operating parameters constantly maintained.

*J_v_* (water flux) was found by calculating the weight variation in the feed solution side:

(1) $$J_{v}=\frac{\Delta V_{feed}}{A_{m}\times \Delta t}=\frac{\Delta m_{feed}/\rho_{feed}}{A_{m}\times \Delta t}$$

where $\Delta V_{feed}$ is the volume change of the FS, $\Delta m_{feed}$ is the weight change of the FS, $\rho_{feed}$ is the density of the FS, Δt is the calculating time interval, and $A_{m}$ is the effective membrane area.

*J_s_* (solute flux) was evaluated by calculating the change in salt concentration in the feed side in accordance with the conductivity variations:

(2) $$J_{s}=\frac{\Delta \left(C_{t}V_{t}\right)}{A_{m}\cdot \Delta t}$$

where $C_{t}$ is the solute concentration of the feed solution in g/L and $V_{t}$ is the feed solution volume in liters at the end of the FO test.

The SRSF was determined as *J_s_*/*J_v_*.

### 2.8. Impact of Varying Concentrations of MPD and B-GQDs during IP Process on the Performance in FO Process

The impact of MPD concentration (1.0, 2.0, and 3.0 wt.%) used during the IP process on the forward osmosis performance (solute flux, water flux, salt rejection, and SRSF) of the TFC membranes was assessed in the AL-FS membrane orientation using a 1.5 M of NaCl DS and a 0.1 M of NaCl FS. The AL-DS mode is more desired as it is less prone to fouling and delivers a more stable water flux [76]. Moreover, the effect of incorporating three distinct wt.% of B-GQDs (0.025, 0.050, and 0.075 wt.%) in the PA layer on the FO performance of the developed membranes was assessed. These tests were performed in the AL-DS and AL-FS modes using a 1.5 M NaCl draw solution and a 0.1 M NaCl feed solution.

### 2.9. Water Content Analysis of the Prepared Membranes

Water content is a major membrane feature that is indirectly related to a membrane’s hydrophilic properties. It is associated with the permeation of membranes and the separation process, and it is strongly related to membrane morphology. The water content of the membranes was analyzed by observing the weight of the membrane sample in dry and wet conditions (Equation (3)).

(3) $$\%\;Water\;content=\frac{W_{wet}-W_{dry}}{W_{wet}}\times 100$$

where $W_{wet}$ is the wet membrane weight (mg) and $W_{dry}$ is the dry membrane weight (mg).

### 2.10. Long-Term Performance Assessment of the TFNC Membranes

The developed TFNC membranes demonstrated a high water flux, so it was not possible to constantly operate these membranes in the FO system in one run. Therefore, 8 continuous runs were performed in the forward osmosis unit with no backwashing of the system. Nearly 108 L of the FS was employed in this long period of study, which lasted for 7.5 h. About 14 L of the 0.1 M sodium chloride FS and 2 L of the 1.5 M sodium chloride DS were employed each time for the long-term FO experiment.

The reversible nature of the fouling layer accumulated on the PA layer was analyzed via physical cleaning for thirty minutes with a higher cross-flow velocity (CFV) (twice the values used for the long-period forward osmosis test). The efficiency in cleaning is presented in Equation (4).

(4) $$R\left(\%\right)=\frac{\left(J_{c}-J_{a}\right)}{\left(J_{b}-J_{a}\right)}$$

where *J_a_* is the water flux (L/m^2^ h) after Run 8, *J_b_* is the water flux (L/m^2^ h) during Run 1 (fresh membrane) (L/m^2^ h), and *J_c_* is the water flux (L/m^2^ h) subsequent to physical cleaning.

### 2.11. Chlorine Resistance Examination of Developed TFC/TFNC Membranes

Chlorine is the most frequent and efficient disinfectant employed in the water treatment process. Due to its corrosive properties, a chlorine attack can cause significantly worsen membrane performance [77,78]. A chlorine attack is a critical challenge that could damage membrane integrity, reduce a membrane’s lifetime, or/and upsurge maintenance expenses [79,80]. In the current study, a 1000 ppm sodium hypochlorite solution was prepared using sodium hypochlorite (NaOCl) and DI water for the chlorine resistance analysis. The pH value of the solution was modified to 5.5 by using solutions of NaOH and HCl. The TFC and TFNC FO membranes to be tested were initially immersed in the prepared NaOCl solution with protection from light at 25 °C, and three immersion times were used, i.e., 2, 4, and 6 h. After each specific period, the membranes were taken from the solution, thoroughly washed using DI water, and subsequently stored in a DI water bath for a minimum of 24 h before the FO experiment. To examine the chlorine resistance properties of the TFC and TFNC membranes, 0.1 M of sodium chloride and 1.5 M of sodium chloride were used as the FS and DS, respectively. During each test, the water flux and SRSF values of the TFC and TFNC membranes were determined to evaluate their chlorine resistance property.

### 2.12. Validation of the FO Experimental Results

In a study carried out by Kim et al. [81], the team demonstrated a stepwise approach for developing a prediction model for scaled-up of FO. In their study, the statistical analysis confirmed that the stepwise model prediction showed a better agreement with experimental results of water flux and salt flux compared with the prediction from a lumped parameter model over a temperature range of 15 °C to 35 °C. The results and analysis from their investigation demonstrated the advantages of the stepwise model for prediction of water flux. In our study, we modified some empirical parameters in the stepwise model to specify the osmotic pressure and to estimate the effect of different membrane parameters (porosity, tortuosity, water permeability, salt permeability and salt rejection).

The water permeability (*A*, LMHB), salt rejection (*R*, %), and salt permeability (*B*, LMH) of the membranes were examined by testing the membranes with a lab-scale dead-end filtration system under the reverse osmosis mode. The experiments were performed at room temperature with an effective membrane area of almost 42 cm^2^. The *A* values were attained from the experiments using deionized water as the feed under an applied pressure of 0.28 bar. Next, 100 mL of water was collected, and the time interval, which was 28 s for TFC membrane, was recorded. The testing was conducted 3 times, and the average water flux (*J_w_*) and water permeability coefficient (*A*) were established using Equations (5) and (6):

(5) $$J_{w}=\frac{\Delta V}{A_{m}\Delta t}=\frac{\frac{\Delta m}{\rho }}{A_{m}\Delta t}$$

(6) $$A=\frac{J_{w}}{\Delta P}$$

where Δ*V*, *A_m_*, Δ*t*, Δ*m*, ρ, and Δ*P* are the permeate volume, effective membrane surface area, time interval, weight variation of the feed solution, feed density, and applied transmembrane pressure, respectively.

The intrinsic separation properties of the TFC and TFNC membranes with respect to salt rejection and the salt permeability coefficient were evaluated using a cross-flow reverse osmosis system where the applied pressure was 0.28 bar and the feed solution was 1.4 M of NaCl. The permeation cell had a total effective membrane surface area of 42 cm^2^. A liquid flow rate of 60 L/h was applied during the testing.

According to solution–diffusion theory, salt rejection (*R*) and salt permeability coefficient (*B*) were determined using Equations (7) and (8), respectively:

(7) $$R=\left(1-\frac{C_{p}}{C_{f}}\right)\times \;100\;$$

(8) $$B=\frac{A\left(1-R\right)\left(\Delta P\right)}{R}$$

where *C_p_* and *C_f_* are the salt concentration in the feed and permeate, respectively; Δ*P* is the applied pressure across the membrane; and *A* is the water permeability calculated in Equation (6).

The structural parameter (*S*) is defined by the tortuosity (τ), thickness (*l*), and porosity (ε) of the porous structure, as shown in the equation below:

(9) $$S=\frac{\tau \times \;l}{\varepsilon }$$

## 3. Results and Discussion

### 3.1. B-GQD Characterization

#### 3.1.1. Morphological Properties Analysis

**Figure 5:**
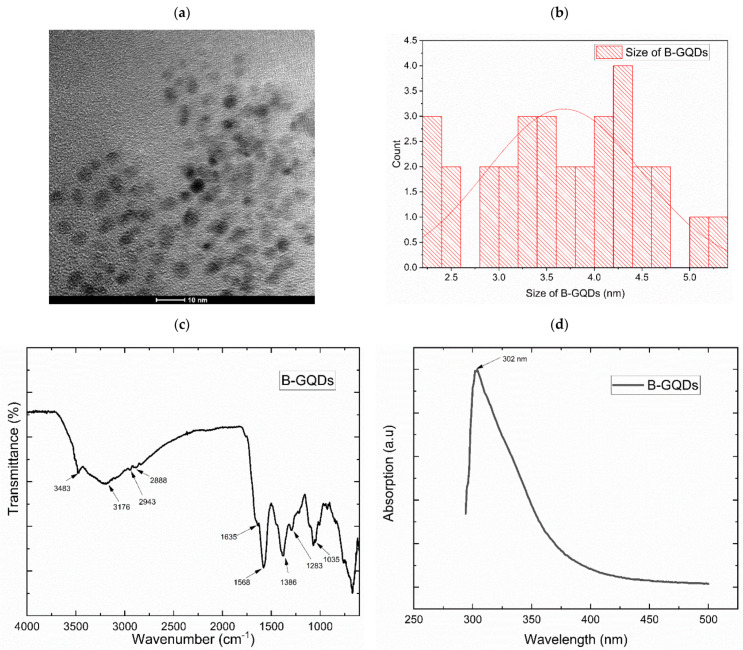

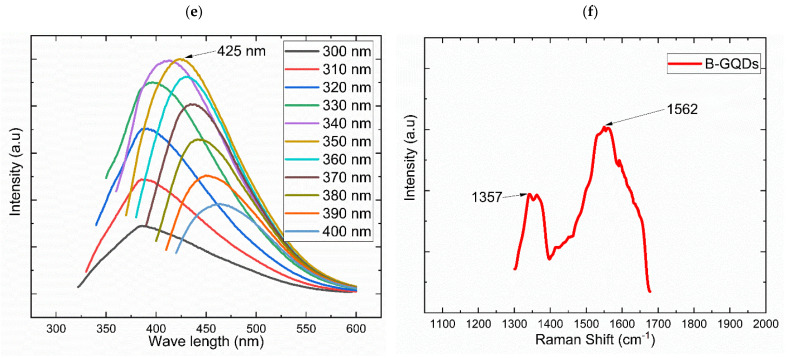
(**a**) Transmission electron microscopy image of B-GQDs; (**b**) the size distribution of B-GQD particles; (**c**) FTIR spectrum of B-GQDs; (**d**) B-GQD Raman spectrum; (**e**) B-GQD UV–vis spectrum; (**f**) PL spectrum of B-GQDs.

A TEM image of the synthesized B-GQDs is presented in Figure 5a. From the obtained image, it can be noted that the developed B-GQDs were spherical and mono-dispersed. Figure 5b presents the particle sizes of different B-GQDs obtained from the particle size distribution data. These histograms were plotted with the Image J software. From the detailed assessment of the structure of B-GQDs, it was confirmed that the B-GQDs had a uniform particle size of less than 5 nm and the B-GQD particles were not aggregated. The prepared B-GQDs showed a narrow size distribution and diameters in the range of 2.5 to 5 nm. The results obtained from the TEM analysis agreed with the images obtained in work conducted by Kumawat and his team members [70]. The formation of the B-GQDs may have been caused by the carbonization of organic precursors during the heat treatment process in the autoclave. The B-GQD material’s carbonization degree could have helped the control of the particle size of the formed nanomaterials. From the analysis of the B-GQD particle sizes, it was noted that the emission properties of the B-GQDs were attributed to the effect of quantum size or the electron/hole recombination in the GQD particles [82]. A smaller particle size and the surface chemistry of GQDs expedite their dispersion in polymeric matrices and polar solvents such as water, which are significant qualities regarding their use in membrane preparation and separation processes [83].

#### 3.1.2. Chemical Compositional Analysis

The successful preparation of GQDs was confirmed by the results of FTIR analysis. The hydrothermally prepared B-GQDs were certainly water-dispersible due to the availability of hydroxyl and carboxylic groups on their surface, which was established by the FTIR examination [84]. As presented in Figure 5c, the peak formed at around 1386 cm^−1^ indicated the existence of –COOH, while the peak seen at about 3483 cm^−1^ arose from the bending vibrations of the O–H bonds. The two peaks observed at approximately 2943 cm^−1^ and 2888 cm^−1^ were related to the C–H bond’s stretching vibrations, and the peak that appeared at almost 1635 cm^−1^ was attributed to the benzene ring vibrations of C = C bonds. Additionally, the peak at 1568 cm^−1^ was due to the C = C stretching vibration of the aromatic ring, which established that the synthesized B-GQDs comprised aromatic rings. Furthermore, the peaks noted at 1283 cm^−1^ and 1035 cm^−1^ could be attributed to the vibrating absorption bands of C–O–C and C–O stretching vibrations in epoxides, respectively. These FTIR results of the B-GQDs were the same as those found by Shi et al. [84].

The UV–vis absorption spectrum of the B-GQDs (Figure 5d) presented a strong background absorption at wavelength of about 302 nm. This could have been because of the aromatic sp^2^ domains’ π–π* transitions [67]. In this UV–vis spectrum analysis, the peak obtained for B-GQDs was the same as the result obtained for GQDs in a study performed by Roy et al. [67], who prepared GQDs from fenugreek and neem leaves that were employed in a white light-emitting diode (LED) application. In our study, the prepared B-GQD aqueous solution exhibited a yellowish-orange color and emitted a blue luminescence under UV light which could have been due to the presence of carbon particles with luminescent properties.

The photoluminescence spectra of the developed B-GQDs are shown in Figure 5e. The proper analysis of the PL spectra of the B-GQDs was carried out, and it was confirmed that at particular excitation wavelengths in the 300–400 nm range, an increment in the emission intensity took place until a maximal emission at 350 nm, and then it decreased. The increase in the excitation wavelengths generated an equivalent decrease in the intensity of PL emission. The maximal photoluminescence intensity of this nanomaterial was shown at 425 nm. The PL assessment of the B-GQDs described in the current research work was consistent with the PL results of GQDs reported in some other works [85,86]. The fluorescence emission mechanism of the prepared B-GQDs was mostly a result of the presence of an aromatic conjugated framework, with emissive free zigzag sites having a carbene-like triplet ground state, quantum size effect, presence of groups with oxygen, and emission traps on the surface [87]. Similar to other carbon-based nanostructured materials with fluorescent properties, the excitation-dependent PL performance of graphene quantum dots is associated with the optical selection of varyingly sized graphene quantum dots, as well as the surface defects present in this nanomaterial [88].

#### 3.1.3. Crystallinity Study

The defects in the carbonic framework caused a rise in the D-band or defect band in the Raman spectrum, as observed at ~1357 cm^−1^ in the developed graphene quantum dots (Figure 5f). The Raman spectra of the synthesized B-GQDs confirmed a G-band-dominated framework, which implied that the developed B-GQDs were more graphene-like. This peak formation was a confirmation of the disorder generated in the crystalline sp^2^ carbon form. Furthermore, a graphitic G-band at ~1562 cm^−1^ indicated the graphitic domains in the structure. The results obtained from the Raman spectrum also established that the D-band was developed due to the disorder present in the sp^2^ hybrid carbon, whereas the G-band was developed due to the graphitic framework. The D-band peak position in the B-GQD spectrum did not depend on the developed quantum dots’ diameter, and the G-band peak location varied with the B-GQD particle size. The I_D_/I_G_ ratio is a significant aspect as a defect density measure in which the quantum dots edges denote the defect sites in graphene of larger area. The high I_D_/I_G_ ratio of GQDs confirms an amorphous nature and a low I_D_/I_G_ ratio confirms a high graphitization degree. In this study, the I_D_/I_G_ ratio of B-GQDs was 0.612, and this result confirmed that the B-GQDs had the structure of nano-crystalline graphite, which is nearly the same as the results reported by Hu, Q. et al. [89] and Shin et al. [90].

### 3.2. Characterization of TFC and B-GQD-Incorporated TFNC Membranes

#### 3.2.1. Membrane Morphological Analysis

**Figure 6:**
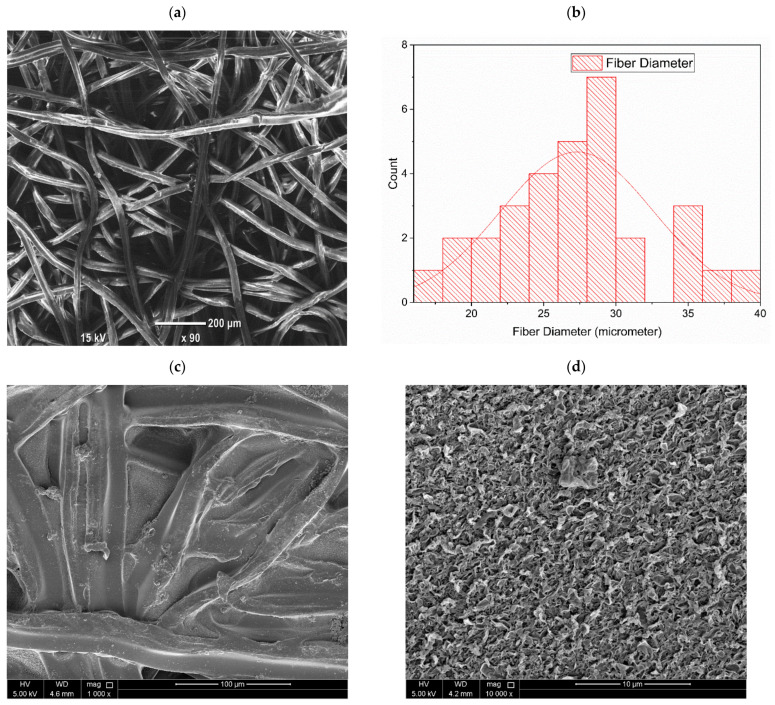

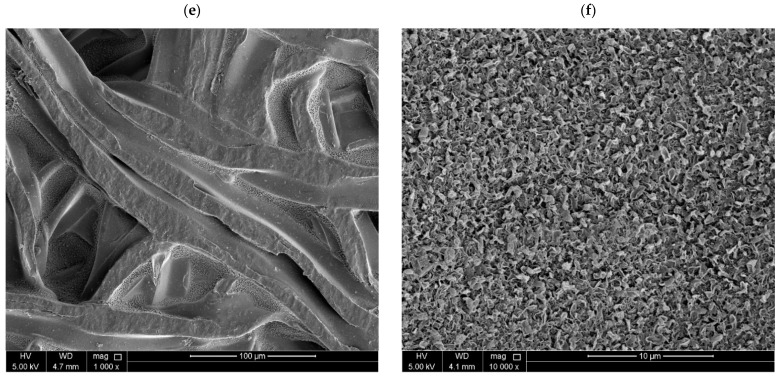
SEM images of (**a**) PES-based NFM substrate layer; (**b**) distribution of nanofiber diameter in NFM substrate; (**c**) topmost surface image of thin-film composite membrane at 1000× magnification; (**d**) topmost surface image of thin-film composite membrane at 25,000× magnification; (**e**) topmost surface image of the 0.05-B-GQD/PA TFNC membrane at 1000× magnification; (**f**) topmost surface image of the 0.05-B-GQD/PA TFNC membrane at 25,000× magnification.

Scanning electron microscopy images of the morphology of the NFM substrate top surface, TFC membranes, and TFNC membranes are shown in Figure 6. Figure 6a shows a scanning electron microscopy image of the NFM, and a uniform fiber network can be detected from the image. In Figure 6b, the distribution of nanofiber diameter in NFM substrate can be observed, and it was found that most of the fibers had diameters ranging from 20 to 30 μm. The PA layer was deposited over the PES NFM substrate via the IP process to form TFC membrane after the heat-press treatment. Figure 6c shows a top-surface image of a TFC membrane at 1000× magnification. It is significant to note that the shapes and patterns of the nanofibers can be clearly seen under the selective PA layer. However, these patterns are typically not observed in TFC membranes with substrate layers prepared via the phase inversion method [91]. On the other hand, a similarly patterned PA layer has noted in some studies where electrospun NFM support was used for TFC membranes [92,93,94]. Our nanofiber membrane demonstrated a good porosity, increased surface roughness, and bigger interstitial pore frames, which probably caused the formation of this type of patterned polyamide layer. Furthermore, Figure 6d shows a top-surface image of the TFC membrane at a magnification of 25,000×. Typical rigid-and-valley structures were observed on the TFC membrane surface, which revealed the successful development of a selective PA layer. Figure 6e displays the top surface image of the 0.05-GQD/PA TFNC membrane at 1000× magnification, whereas Figure 6f shows a zoomed-in image of this membrane at a magnification of 10,000×. Moreover, it was observed that the B-GQDs were not visible on the polyamide layer surface, suggesting the proper inclusion of the developed nanomaterial in the active layer.

**Figure 7:**
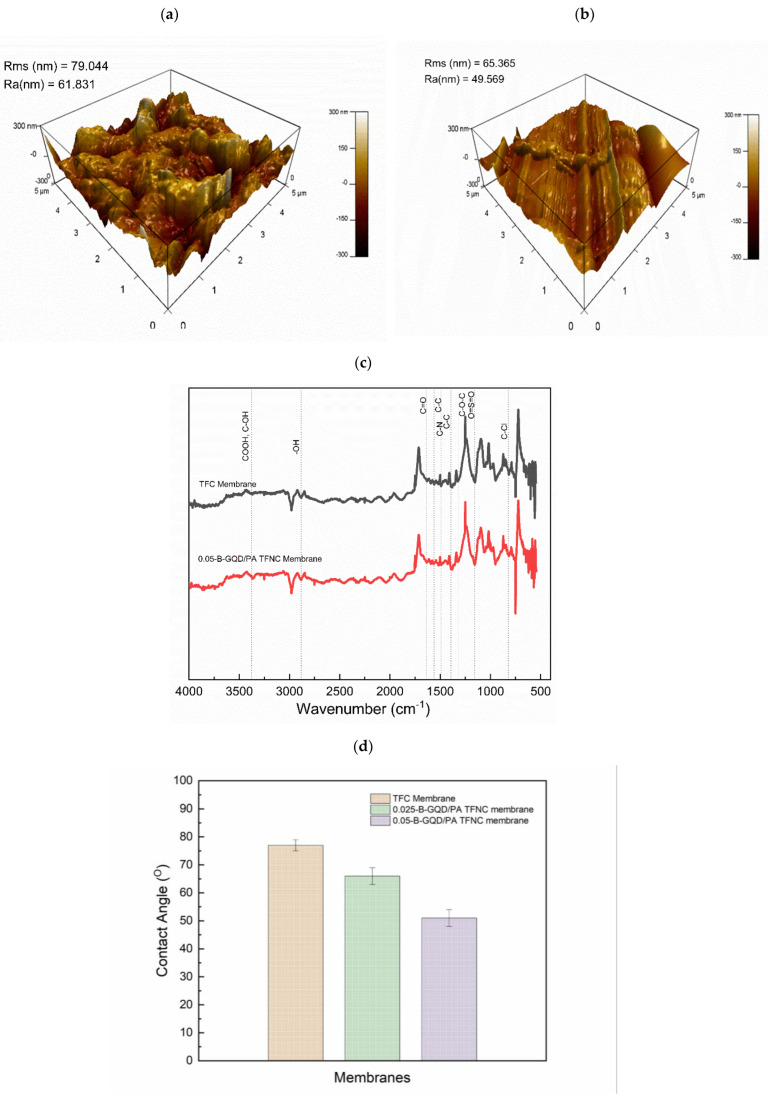
AFM images of (**a**) TFC NFM substrate; (**b**) 0.05-B-GQD/PA TFNC membrane; (**c**) FTIR spectrum of TFC and 0.05-B-GQD/PA TFNC membranes; (**d**) CA analysis of TFC membrane, 0.025-B-GQD/PA TFNC membrane sample, and 0.05-B-GQD/PA TFNC membrane sample.

AFM analysis was carried out to analyze the surface morphologies of the TFC and TFNC membranes. AFM images of the membranes are shown in Figure 7a,b. The mean roughness (R_a_) and root-mean-squared roughness (R_ms_) of the TFC and TFNC samples are also presented in Figure 7a,b. The Rms value of the 0.05-B-GQD/PA TFNC membrane improved by 17.3% compared with the TFC membrane. Additionally, the Ra value of the 0.05-B-GQD/PA TFNC membrane improved by 19% relative to the TFC membrane. Thus, it was confirmed that the nanocomposite membrane incorporated with B-GQDs exhibited a smoother surface than the pristine TFC membrane. When integrated into the selective layer of the TFC membranes, the GQDs could form uniform distributions in a manner where the GQDs’ stretch in the PA layer was extremely small. As a result, a smooth surface was observed for the nanocomposite membrane. With the inclusion of the developed nanomaterial, the surface roughness of the nanocomposite membrane diminished. Furthermore, a small particle size of GQDs may result in the formation of a thin selective PA layer. Therefore, an appropriate dispersion of GQDs could effectively contribute the successful formation of a polyamide layer via the interfacial polymerization process [95,96]. Seyedpour et al. [56], who fabricated nanocomposite membranes with GQDs synthesized from citric acid, reported that the nanocomposite membrane demonstrated a good surface smoothness. This surface smoothness of the GQD-incorporated nanocomposite membranes was due to two reasons. Primarily, the diffusion of MPD mostly produced a ridge and valley framework; however, the horizontally-oriented GQDs hindered the penetration of MPDs to the organic phase, which resulted in smooth surface formation. Additionally, the functional groups existing in the GQDs establish surface interactions with TMC and MPD monomers during the interfacial polymerization process, thereby influencing the rate of reaction of MPD and TMC [97].

#### 3.2.2. Membrane Compositional Analysis

**Figure 8:**
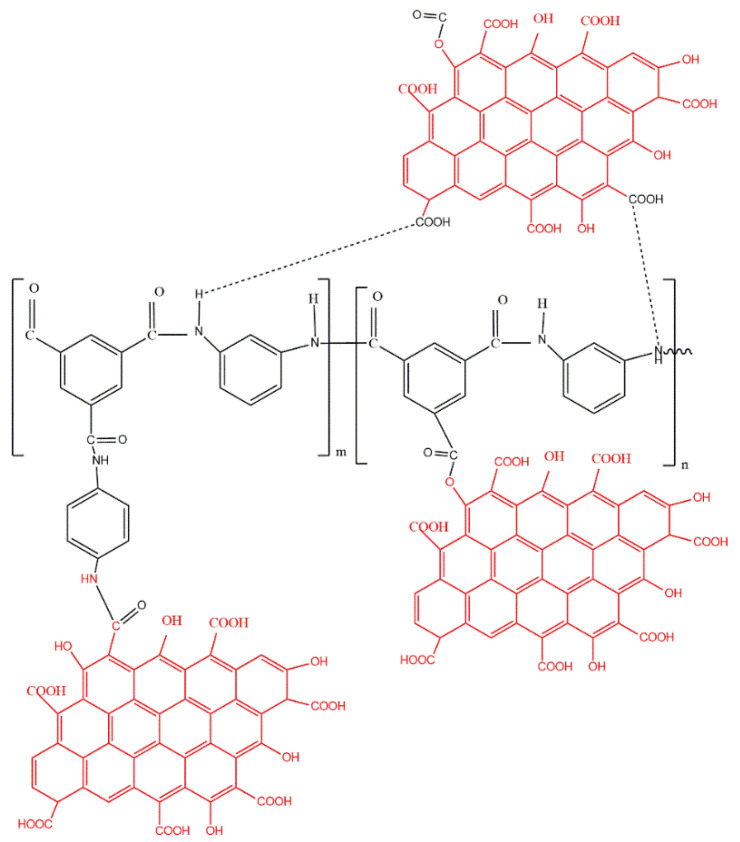
Interaction mechanism of PA chain and GQDs, where m and n represent the crosslinked and the linear fractions of the PA framework, respectively (m + n = 1).

The formation of the selective PA layer was confirmed via the Fourier transform infrared spectra of the pristine thin-film composite and GQD-based TFNC membranes, as presented in Figure 7c. The peak at 815 cm^−1^ confirmed the C–Cl stretching vibrations of the unreacted acid chloride groups. Furthermore, the asymmetric and symmetric O=S=O stretching vibrations of the polyether sulfone layer were confirmed by the peaks developed at 1156 and 1310, respectively. Additionally, the peaks at 1481 and 1389 demonstrated the availability of C–C in the aromatic rings [56,98]. The amide carbonyl stretching vibrations were revealed by the peak seen at about 1635 cm^−1^. Furthermore, the interactions of in-plane N–H amide II bending vibrations and C–N stretching were confirmed by the band at about 1563 cm^−1^ [99,100]. However, it was noted that in the 0.05-B-GQD/PA TFNC membrane, with the incorporation of B-GQDs into the selective layer, the intensity of the main peaks substantially decreased, and some of the peaks just completely disappeared due to the presence of GQDs [101], demonstrating GQD-PA layer interactions [102]. Moreover, the enhanced stretching vibrations of carbonyl bonding in the 0.05-B-GQD/PA TFNC membrane confirmed the development of the amide linkages by the interactions between carboxyl groups of GQDs and amine groups present in MPDs [103]. Furthermore, the peak formed at 1747 cm^−1^ of the 0.05-B-GQD/PA TFNC membrane was due to the stretching vibrations of carbonyl in the ester groups formed via the interactions between the GQDs’ functional groups and the PA layer’s carboxylic groups (Figure 8) [104]. In relation to the TFC membrane, the 0.05-B-GQD/PA TFNC membrane presented a higher intensity peak at about 3360 cm^−1^ that confirmed the stretching vibrations of B-GQDs’ hydroxyl functional groups, which could enhance the nanocomposite membrane’s surface hydrophilicity, as demonstrated by the contact angle results of thin-film composite and TFNC membranes [105]. The FTIR results obtained in the current study are in agreement with those previously reported by Seyedpour et al. [56].

#### 3.2.3. Membrane Hydrophilicity Analysis

Figure 7d shows the CA values of the TFC and TFNC membranes. It was confirmed that the addition of B-GQDs significantly improved the hydrophilic properties of the selective PA layer. The CA of the membranes reduced from 77° (for thin-film composite membrane) to 66° (0.025-B-GQD/PA TFNC membrane) and 51° (0.05-B-GQD/PA TFNC membrane). The results from the FTIR analysis of the nanocomposite membranes confirmed that the hydroxyl-functional groups’ stretching vibrations were available in the B-GQDs, and this generated improved surface hydrophilic properties [105]. The CA value of the TFNC membranes decreased by 34% relative to the thin-film composite membranes. This substantial development in the surface hydrophilicity of the B-GQD-based TFNC membrane could have been due to the availability of functional groups with ample oxygen-consisting groups on the B-GQD surface [100]. Improving surface hydrophilic properties is an appropriate method to reduce the adhesion of fouling agents and therefore improve fouling resistance [103,106]. The contact angle values of the thin-film composite membrane and the TFNC membrane obtained in this study followed the same decreasing trend as that noted for membranes in a study performed by Seyedpour et al., where GQDs prepared via the direct pyrolysis of citric acid were employed [56].

**Figure 9:**
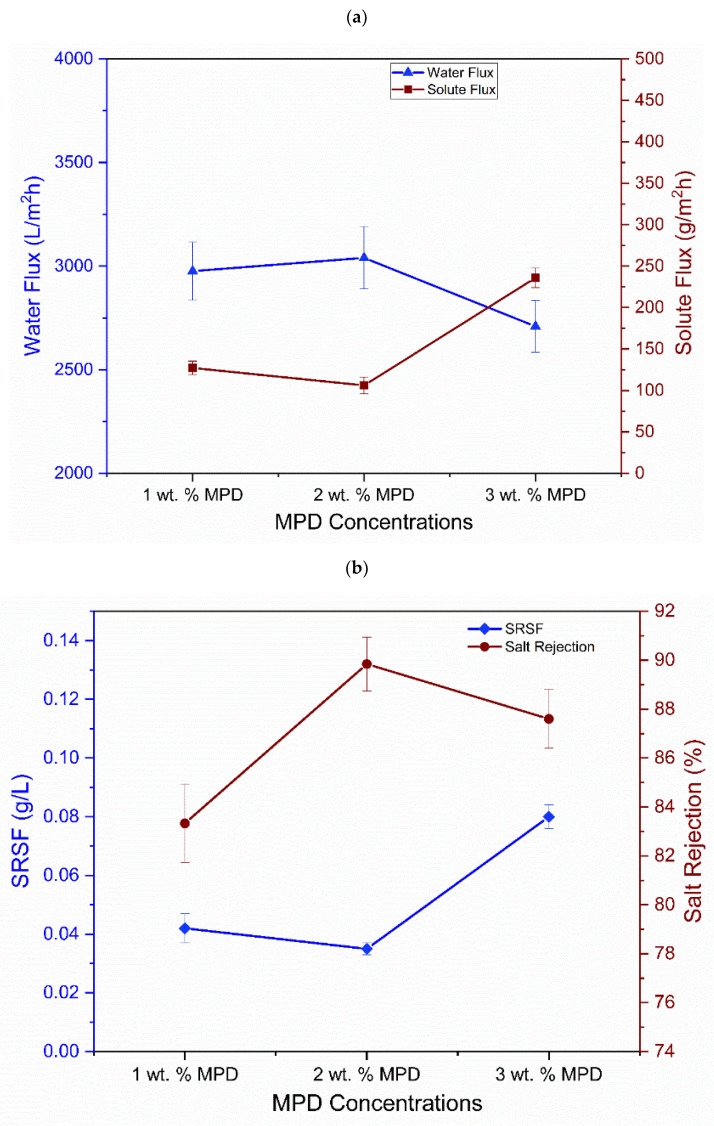

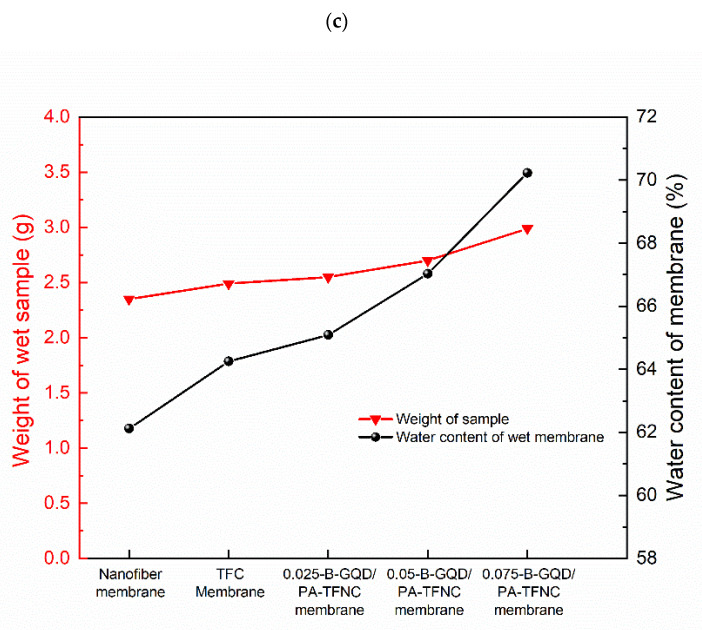
(**a**) Effect of MPD concentration on the water flux and solute flux of the TFC-FO membrane; (**b**) effect of MPD concentration on the SRSF and salt rejection of the TFC-FO membrane; (**c**) water content assessment of TFC and TFNC membranes.

Table 1 presents the water content analysis of the TFC and TFNC membranes. It can be noted from Figure 9c that the rise in water content was directly proportional to the increase in the concentration of B-GQDs. For the membrane used for B-GQD incorporation, the water content of the base nanofiber membrane was 62% and increased to 64.25% after the formation of the PA layer by the IP process. Meanwhile, the 0.025-B-GQD/PA TFNC membrane demonstrated a water absorption of 65.09%. Furthermore, the 0.050-B-GQD/PA TFNC and 0.075-B-GQD/PA TFNC membranes showed water contents up to 67.03 % and 70.23%, respectively. This rise in water content was due to the increase in void volume, which led to the development of a larger pore size framework and consequently enhanced the water uptake in the pores [107].

### 3.3. FO Membrane Performance of TFC and TFNC Membranes

The effects of incorporating varying concentrations of B-GQDS into the selective layer of the TFC membrane on the forward osmosis membrane performance (solute flux, water flux, salt rejection, and specific reverse solute flux) were investigated. Moreover, the long-period performance evaluation and chlorine resistance analysis of the developed TFNC membranes were performed.

#### 3.3.1. Impact of MPD Concentration in TFC on FO Membrane Performance

The effect of three different MPD concentrations (1 wt.%, 2 wt.%, and 3 wt.%) on the performance of the TFC-FO membranes with respect to water flux, solute flux, SRSF, and salt rejection was tested in the AL-FS mode while employing 1.5 M of sodium chloride as the draw solution and 0.1 M of sodium chloride as the feed solution. As demonstrated in Figure 9a, with the increase in the concentration of MPD from 1.0 wt.% to 2.0 wt.%, the TFC-FO membrane’s water flux initially increased (from 2976 L/m^2^ h to 3040 L/m^2^ h). Subsequently, with the further increase in the concentration of MPD to 3 wt.%, the water flux reduced to 2709 L/m^2^ h. Thus, the maximum water flux of 3040 L/m^2^ h was accomplished at an MPD concentration of 2.0 wt.%. Regarding the solute flux of the membrane, it was noted that the solute flux initially decreased and subsequently increased, attaining a minimal value of 106 g/m^2^ h at the 2.0 wt.% MPD concentration. Moreover, with the increase in the concentration of MPD from 1.0 wt.% to 2.0 wt.%, the TFC-FO membrane’s salt rejection initially increased and subsequently reduced, and the maximal salt rejection of 89.84% was accomplished at the 2.0 wt.% MPD concentration (Figure 9b). A high *J_s_*/*J_v_* ratio is not recommended due to the following reasons: (i) an extreme accumulation of salt on the feed side resulting from reverse salt diffusion, (ii) a high exposure of the reverse salt diffusion-produced fouling of the membrane, (iii) a reduced retention against contaminants present in the FS, and (iv) an increased replacement expenses of the DS. It can be observed that the rise in MPD concentration from 1 wt.% to 2 wt.% enhanced the PA layer’s cross-linking degree, leading to an increased selectivity and an improved water flux of the TFC membrane in the FO process. Moreover, a membrane with improved rejection will have a lower *J_s_*/*J_v_* value [108]. Nevertheless, the excessive 3 wt.% concentration of MPD led to the development of a dense PA layer with a reduced water flux [109]. Thus, 2.0 wt.% of MPD was identified as the optimized monomer loading and was used for the preparation of the TFNC membranes and for further FO performance evaluation. The proper explanation for the higher flux values in the FO membranes with SBS nanofiber support was explained in a study by Saleem et al. [30]. Here, the membrane’s nanoporous structure was an extremely significant factor that regulated its water flux and the rejection of solutes. Furthermore, the greater surface volume ratio of the solution blown-spun fibers was a main reason for the extremely high water flux. Moreover, because of the bigger pore size relative to other studied membranes, the flow of accumulated/bulk hydrated water molecules was allowed, which was another main reason for the super high water flux.

#### 3.3.2. Result of Varying B-GQD wt.% on the FO Performance

**Figure 10:**
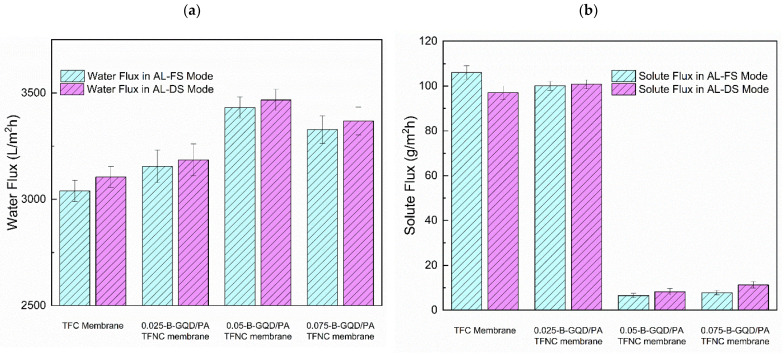

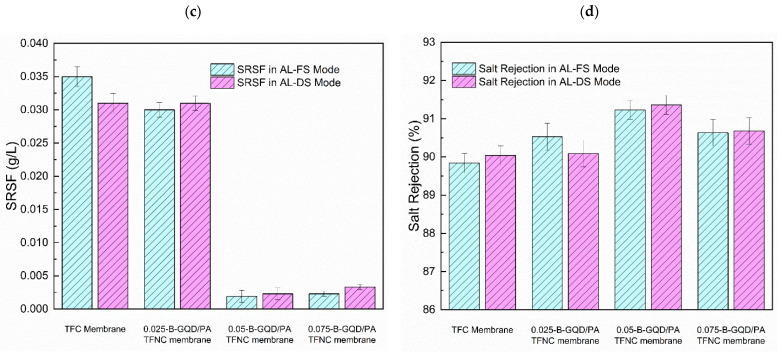
(**a**) Water flux of the TFC and TFNC membranes in the AL-DS and AL-FS orientations at varying B-GQD concentrations; (**b**) salt flux of the TFC and TFNC membranes in the AL-DS and AL-FS membrane orientations at varying B-GQD concentrations; (**c**) SRSF of the TFC and TFNC membranes in the AL-DS and AL-FS membrane orientations at varying B-GQD concentrations; (**d**) salt rejection of the TFC and TFNC membranes in both membrane orientations at varying B-GQD concentrations.

The impact of incorporating varying concentrations of B-GQDs on the forward osmosis membrane performance (solute flux, water flux, SRSF, and salt rejection) of the TFNC membranes was assessed in the AL-DS and AL-FS orientations using the 0.1 M sodium chloride FS and 1.5 M sodium chloride DS, and the results are shown in Figure 10.

From the forward osmosis performance evaluation, it was found that all B-GQD-added nanocomposite membranes demonstrated a lower solute flux, a higher water flux, a slightly higher salt rejection, and a reduced SRSF relative to the TFC membranes. Figure 10a shows the effect of incorporating varying concentrations of B-GQDs on the water flux in the AL-DS and AL-FS orientations. In the AL-FS orientation, the TFC membrane’s water flux was observed to be 3040 L/m^2^ h. With the addition of B-GQDs into the PA layer, the membrane’s water flux increased with 0.025 wt.% of B-GQDs (3155 L/m^2^ h) and 0.050 wt.% of B-GQDs (3432 L/m^2^ h), and it subsequently reduced with 0.075 wt.% of B-GQDs (3328 L/m^2^ h). Consequently, an increment of 3.8%, 13.0%, and 9.4% in water flux was achieved by the addition of 0.025, 0.050, and 0.075 wt.% of B-GQDs, respectively, relative to the water flux of the thin-film composite membrane. These increments were mainly caused by the water channels established between the active polyamide layer and the developed graphene quantum dots, which offered extra channels for water penetration. The interfacial gaps between the PA framework and the B-GQD nanosheets created nanochannels that allowed for the transport of water molecules [56]. Moreover, more driving force generated from the hydrogen-bonding interactions between the water molecules and hydroxyl/carboxyl groups of B-GQDs sped up the water molecules that penetrated the water channels. The above-mentioned statements confirmed that the B-GQD incorporation into the selective layer enhanced the surface hydrophilic properties of the membrane by offering a quick transfer of water molecules across the membrane [56,100]. This improvement in surface hydrophilicity was confirmed by the CA examination results. However, it was found that the water flux declined at a higher loading of B-GQDs, i.e., with the incorporation of 0.075 wt.% of B-GQDs into the TFC membrane. This might have been because of the B-GQD accumulation, which did not contribute enough nanochannels and led to pore blocking on the surface of the substrate [110]. At excess concentrations, the B-GQDs could also lead to extremely tortuous and prolonged transfer pathways for water molecule transport inside the active PA layer. Accordingly, when the hydraulic resistance increased, a reduction in water flux followed. It was observed that all the nanocomposite membranes demonstrated increased water flux values in the AL-DS membrane orientation compared with that in the AL-FS orientation because the ICP effect in the AL-DS membrane orientation was concentrative and weaker than the AL-FS membrane orientation [111]. The 0.05-B-GQD/PA TFNC membranes demonstrated maximal flux in both the AL-DS (3467 L/m^2^ h) and AL-FS (3432 L/m^2^ h) membrane orientations. For the 0.05-B-GQD/PA TFNC membrane, there was an increase of nearly 11.65% in water flux in the AL-DS orientation and a 12.8% increment in the AL-FS orientation compared with the TFC membrane values.

On the other hand, with respect to the salt flux of the B-GQD/PA TFNC membranes, it was observed that the salt flux value was reduced by the addition of B-GQDs. Figure 10b shows the effect of incorporating diverse concentrations of B-GQDs on the solute flux of the TFC and TFNC membranes in both membrane orientations. The 0.05-B-GQD/PA TFNC membranes exhibited a minimal solute flux of about 93.8 % in the AL-FS orientation, a 91.5% reduction in the AL-DS membrane orientation, and a 91.5% reduction in the AL-DS membrane orientation. In the 0.05-B-GQD/PA TFNC AL-FS membrane orientation, the solute flux attained a reduced value of 6.47 g/m^2^ h compared with the other membranes developed in this study. Nevertheless, the 0.075-B-GQD/PA TFNC membrane showed a minimal increment in solute flux relative to the 0.050-B-GQD/PA TFNC membrane. Moreover, the specific reverse solute flux (*J_s_*/*J_w_*) is a crucial measure in the FO process, indicating the performance of a membrane in relation to selectivity and productivity. Principally, a membrane with a reduced SRSF value can offer better selectivity for the rejection of a solute compared with water. Figure 10c shows the effect of incorporating varying wt.% of B-GQDs on the SRSF of the TFC and TFNC membranes in the AL-DS and AL-FS membrane orientations. In this study, the 0.05-B-GQD/PA TFNC membrane demonstrated reduced SRSF values (0.0023 g/L in the AL-DS membrane orientation and 0.0019 g/L in the AL-FS membrane orientation), and these values were superior to those of some other FO membranes [112,113,114]. With the increase in the B-GQD concentration, it was observed that the specific reverse solute flux initially decreased, and it attained a minimum value with the 0.05 wt.% GQD addition. Subsequently, the SRSF value marginally increased with 0.075 wt.% of B-GQDs, which proved the reverse trend of the solute rejection of the thin-film composite membranes. In a research work performed by Xu and co-workers [57], a similar FO performance was observed. Overall, it was noticed that the solute flux and SRSF of the 0.05-B-GQD/PA TFNC membrane demonstrated a substantial reduction compared with that of the TFC membrane.

Figure 10d presents the effect of incorporating varying amounts of B-GQDs on the salt rejection of the TFC and TFNC membranes in the AL-DS and AL-FS orientations. It was observed in this study that the salt rejection of the 0.025-B-GQD/PA TFNC, 0.050-B-GQD/PA TFNC and 0.075-B-GQD/PA TFNC membranes showed a slight increase compared with the thin-film composite membrane. Nevertheless, the 0.075-B-GQD/PA TFNC membrane exhibited a salt rejection value smaller than that of the 0.050-B-GQD/PA TFNC membrane. This confirmed that the excess B-GQDs had a negligible effect on the salt rejection due to the accumulation and the irregular distribution of B-GQDs inside the selective layer [56,100]. The findings confirmed that the water flux and salt rejection of the B-GQD-incorporated nanocomposite membranes could be increased and that the solute flux and SRSF could be reduced by the addition of an appropriate quantity of B-GQDs into the active layer of the membranes. These GQD-based membranes could also increase the membrane surface hydrophilicity by facilitating the transport of water molecules across the membrane. The 0.05-B-GQD/PA TFNC membrane exhibited improved water flux, lower solute flux, minimal specific reverse solute flux, and marginally enhanced salt rejection compared with the thin-film composite membrane.

During the membrane preparation stage, when the B-GQDs were dispersed in the m-phenylenediamine solution, these GQDs could interact with the TMC and MDP monomers during the interfacial polymerization process, enabling the appropriate integration of B-GQDs into the selective layer of the membrane. Despite the hydrogen bonding, the amine functional groups existing in the m-phenylenediamine monomers might have reacted with B-GQDs and formed new amide bonds in the course of the ultrasonication of the m-phenylenediamine solution. Furthermore, the development of ester and anhydride linkages might have taken place as a result of the interaction between the acid chloride groups of TMC and functional groups of B-GQDs [104]. The presence of ester and anhydride linkages was confirmed from the FTIR results of the membranes. The unreacted acid chloride groups of trimesoyl chloride may have undergone reactions with the carboxyl groups of B-GQDs during the interfacial polymerization process. Otherwise, the hydrogen bonds may have formed via the interactions between the primary/secondary amines and the functional groups of the B-GQDs. Additionally, the covalent bonding between the B-GQDs and the carboxyl groups in the linear fraction of the PA layer might have occurred via condensation reactions. Furthermore, the steric hindrance of the developed nanomaterial reduced the penetration of MPDs and hindered the establishment of the active layer of the membrane [58]. The mass transfer resistance noticeably diminished with the decrease in the PA layer thickness and thus caused a rise in the water flux [97]. Nevertheless, by utilizing a higher concentration of the developed nanomaterial, the whole thickness of the polyamide layer of the TFNC membranes increased, and a denser layer compared with that of the thin-film composite membranes was generated as a result of the MPD solution’s higher viscosity, which caused surface blockage and a considerable decrease in the water flux of the membrane. On the other hand, the increased concentration of the GQDs in the TFNC membranes resulted in GQD leak from the active layer and serious accumulations, along with a thicker selective layer [56].

#### 3.3.3. Long-Period Performance Assessment of the Developed Membrane

**Figure 11:**
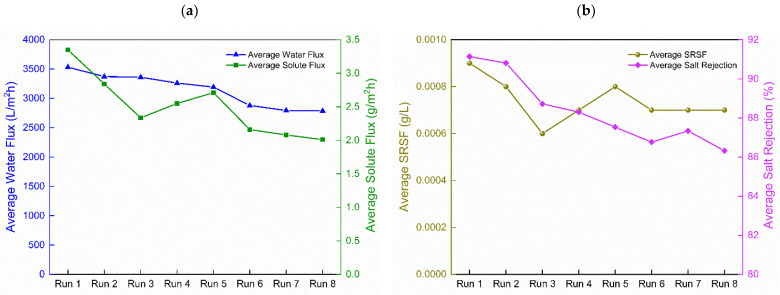

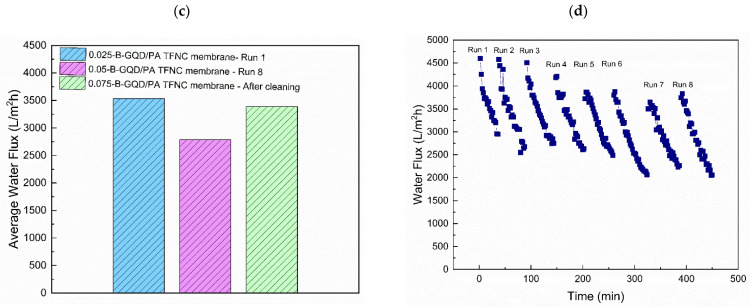
(**a**) Average water flux and average solute flux of the 0.05-B-GQD/PA TFNC membrane in a long-period performance assessment study; (**b**) average specific reverse solute flux (g/l) and salt rejection (%) of the 0.05-B-GQD/PA TFNC membrane in a long-period performance assessment study; (**c**) cleaning effectiveness of the 0.05-B-GQD/PA TFNC membrane after long-period FO experiments; (**d**) water flux (L/m^2^ h) of the 0.050-GQD/PA TFNC membranes in long-period performance analysis—eight runs.

For the long-period performance assessment, only the 0.050-B-GQD/PA TFNC membrane was selected for operation due to its superior FO performance than the other membranes. It showed improved water flux and salt rejection and decreased SRSF and solute flux relative to other membranes. Figure 11d presents the long-duration water flux performance assessment of the 0.05-B-GQD/PA TFNC membrane employed in the forward osmosis process. From the graph, it can be observed that an increased water flux value was noted at the beginning of the FO run, and it subsequently decreased with time. This decrease in water flux during the experiment was affected by the DS dilution and the FS concentration [115]. A similar flux decline behavior over time was also reported by Ali et al. [116] and Zhao et al. [117]. The negative impacts of internal concentration polarization and membrane fouling could have reduced the osmotic water flux and improved the mass transfer resistance as the FS became extremely concentrated due to the water permeation from the FS to the DS and reverse solute diffusion from the DS to the FS [117]. As shown in Figure 11a, the average water flux in the initial run was 3533 L/m^2^ h, and it reduced as the test proceeded before finally reaching a value of 2787 L/m^2^ h during the eighth run.

Figure 11a shows that the salt flux of the 0.05-B-GQD/PA TFNC membrane steadily reduced over time. The salt flux in run 1 was 3.35 g/m^2^ h, and it decreased to 2.83 g/m^2^ h in run 2 and 2.01 g/m^2^ h in run 8. In the same way, a slight decrease was also observed for SRSF, as it was 0.0009 g/L in run 1 and became 0.0007 g/L in run 8 (Figure 11b). This SRSF behavior with time was marginally affected by the reduction in water flux. Figure 11b presents the salt rejection of the 0.05-B-GQD/PA TFNC membrane as a function of time throughout the long-period FO test. The salt rejection of the 0.05-B-GQD/PA TFNC membrane reduced from 91.13% to 86.32% after 7.5 h of FO operation.

After performing the cleaning operation for 1 h, it was observed that the water flux recovered to 80.5% of the initial water flux value. Due to the fact that FO is not a pressure-driven process, the formation of the boundary layer was less compact. Therefore, the lightly deposited foulants could be effortlessly removed by using a high CFV backwashing. Despite the reduction in water flux, the water flux recovery of 80.5% of the original water flux value was accomplished after the fouling-cleaning cycle. This could have be due to the improved hydrophilicity of the 0.05-B-GQD/PA TFNC membrane. Figure 11c demonstrates the cleaning effectiveness after the long-duration FO experiment using the 0.05-B-GQD/PA TFNC membrane. It was noted that the cleaning process was very effective, as the average water flux of the prepared TFNC membrane was almost recovered upon cleaning. Thus, it was confirmed that the forward osmosis performance was practically reversible following the proper cleaning of the nanocomposite membranes despite long-duration FO test runs.

#### 3.3.4. Chlorine Resistance Study of the TFC/TFNC Membranes Developed

The chlorine resistance capability of the TFC, 0.025-B-GQD/PA TFNC, and 0.05-B-GQD/PA TFNC membranes was examined using a 1000 ppm sodium hypochlorite solution with a 5.5 pH value and three immersion times, i.e., 2, 4, and 6 h. The chlorine resistance properties of the TFC and TFNC membranes were examined, and the water flux and SRSF values of the membranes were determined.

**Figure 12:**
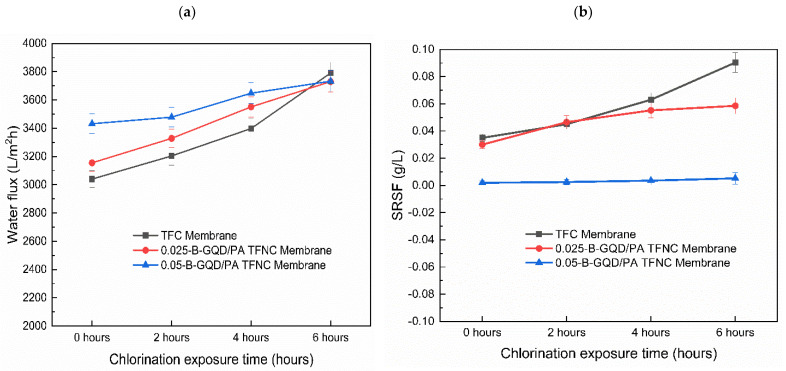
(**a**) Impact of chlorination conditions on the water flux of the TFC and TFNC membranes treated with a 1000 ppm NaOCl solution for 2–6 h at pH 5.5; (**b**) effect of chlorination conditions on the SRSF of TFC and the B-GQD TFNC membranes treated with a 1000 ppm NaOCl solution for 2–6 h at pH 5.5.

**Figure 13:**
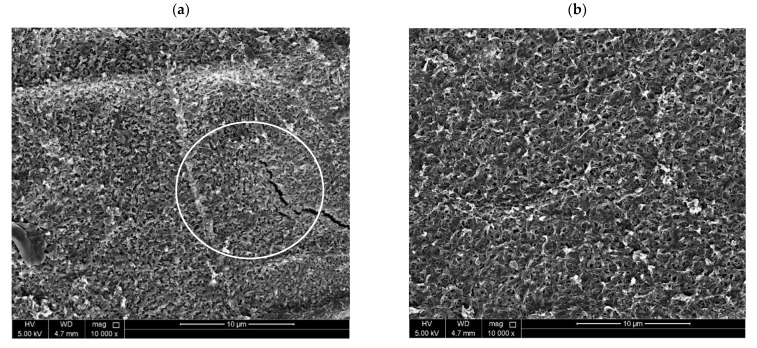
(**a**) SEM image of the TFC membrane treated with a 1000 ppm NaOCl solution for 2–6 h at pH 5.5; (**b**) SEM image of the 0.050-B-GQD/PA TFNC membrane treated with a 1000 ppm NaOCl solution for 2–6 h at pH 5.5.

Due to the fact that the 0.075-B-GQD/PA TFNC membrane demonstrated a decline in forward osmosis performance with respect to water flux, salt flux, salt rejection and SRSF relative to the 0.050-B-GQD/PA TFNC membrane, it was not employed in the chlorine resistance analysis. Figure 12a presents the effect of chlorination conditions on the water flux of the TFC and B-GQD TFNC membranes treated with a 1000 ppm NaOCl solution for 2–6 h at pH 5.5. In the case of the TFC membranes, the amide bonds present in the PA layer of the TFC membrane were susceptible to free chlorine and could be simply destroyed by the direct ring chlorination reactions, Orton rearrangement, and N-chlorination, resulting in a rise in the PA layer free volume and flexibility of the polymer matrix. However, the incorporated B-GQDs could have preserved the underlying PA chains from active chlorine attack. The increased chlorine resistance of the 0.025-B-GQD/PA TFNC and 0.050-B-GQD/PA TFNC membranes may have been due to the hydrogen bonding between the B-GQDs and the PA layer, which prevented the substitution of amidic hydrogen with active chlorine [118]. Consequently, a slow increase in the water flux and SRSF due to the slow degradation of the PA layer was observed for the 0.050-B-GQD/PA TFNC membrane compared with the 0.025-B-GQD/PA TFNC and TFC membranes. The chlorine resistance of the membranes followed the order of the 0.050-B-GQD/PA TFNC membrane > 0.025-B-GQD/PA TFNC membrane > TFC membrane. As observed in Figure 13a, cracks and defects were noted in TFC membrane, confirming that the PA chains were extremely disrupted. On the other hand, the 0.05-B-GQD/PA TFNC membrane demonstrated a comparatively uniform morphology, and the standard structures of PA could still be observed (Figure 13b).

Figure 12b presents the SRSF of the TFC and B-GQD TFNC membranes treated with a 1000 ppm NaOCl solution for 2–6 h at pH 5.5. It was observed that the SRSF values of the TFC and TFNC membranes treated with an acidic NaOCl solution (pH = 5.5) gradually increased over chlorination time. The surging rise of SRSF was avoided due to the compact and dense PA layer structure [118]. The SRSF of the membranes under chlorination exposure followed the order of the TFC membrane > 0.025-B-GQD/PA TFNC membrane > 0.050-B-GQD/PA TFNC membrane. The rate of increase in SRSF values were maximum for the TFC membrane and minimum for the 0.05-B-GQD/PA TFNC membrane. Free chlorine mostly exists in the form of hypochlorous acid (HOCl) in acidic solutions [119], which results in N-chlorination by changing the polar N-H bonds of amide groups on PA layers to N-Cl bonds of less polarity [120]. The reaction of ring-chlorination happened later via Orton rearrangement on the selective layer surface, with the membrane additionally exposed to the HOCl solution [121], which allowed free chlorine to eventually graft to the PA layer’s benzene rings. Accordingly, the crosslinking network of the PA surface developed on the basis of chemical bonds and the internal hydrogen bonds was damaged, which deteriorated the selectivity of the membrane, resulting in a rise in the SRSF value. Similar results for the water flux and SRSF values were stated by Meng et al. [79] and Song et al. [58]. Thus, it was confirmed that the 0.050-B-GQD/PA TFNC membrane showed a much slower rate of PA layer damage while exposed to free chlorine and therefore possessed good chlorine resistance characteristics. For the 0.050-B-GQD/PA TFNC membrane, slow and mild increments in FO water flux and SRSF were noted during the chlorine resistance analysis.

According to the FO performance analysis (solute flux, water flux, SRSF, and salt rejection), chlorine resistance analysis, and long-term performance assessment, the 0.05-B-GQD/PA TFNC membrane was the most suitable developed FO membrane of the various studied membranes.

#### 3.3.5. Validation Study of the FO Experimental Results

**Figure 14:**
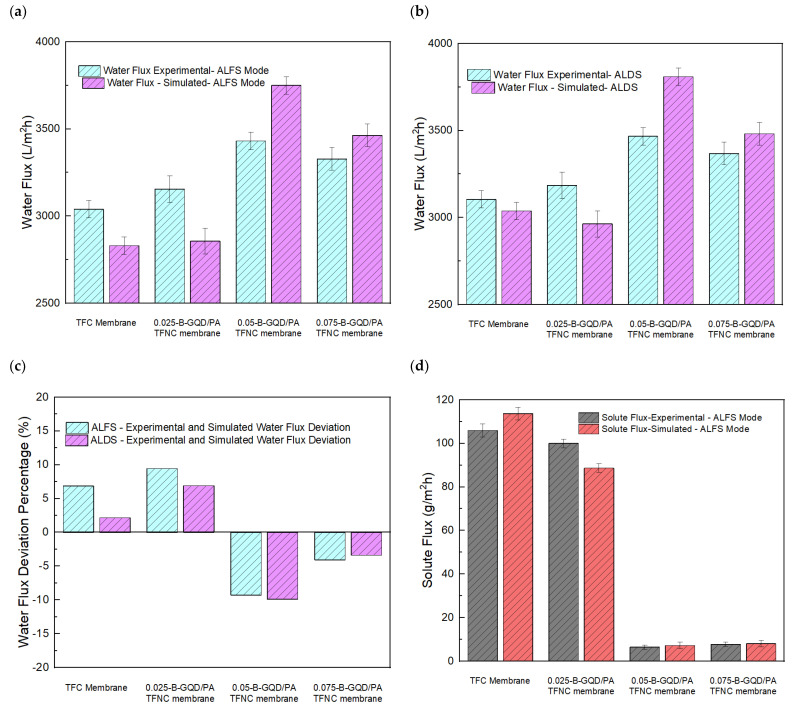

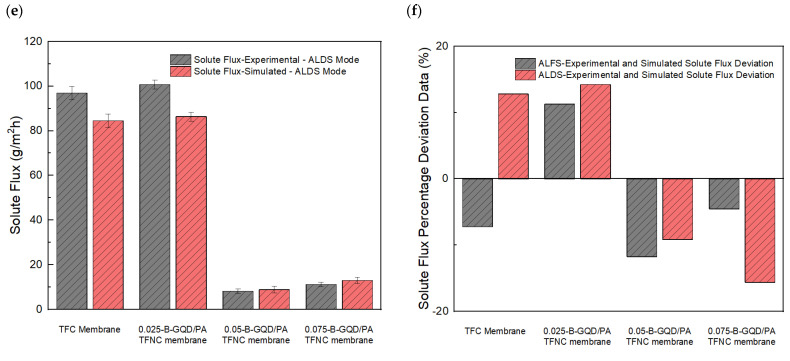
(**a**) AL-FS mode experimental and simulated water flux; (**b**) AL-DS mode experimental and simulated water flux; (**c**) AL-FS and AL-DS mode experimental and simulated water flux percentage deviation; (**d**) AL-FS mode experimental and simulated solute flux; (**e**) AL-DS mode experimental and simulated solute flux; (**f**) AL-FS and AL-DS mode experimental and simulated solute flux percentage deviation.

A modeling study of the TFC membrane, 0.025-B-GQD/PA TFNC membrane, 0.05-B-GQD/PA TFNC membrane, and 0.075-B-GQD/PA TFNC membrane was performed. Table 2 presents an evaluation of the membrane-intrinsic properties from the RO system. Figure 14a,b shows the experimental and simulated water flux of the TFC and TFNC membranes in the AL-FS and AL-DS modes, respectively. Moreover, Figure 14c presents the experimental and simulated water flux percentage deviation in both the AL-FS and AL-DS modes. It was noted that the 0.050-B-GQD/PA TFNC membrane demonstrated a water flux percentage deviation of −9% in both the AL-DS and AL-FS modes. This confirmed that the water flux was greater in the simulation study than in the experimental study. This trend was the same for the 0.075-B-GQD/PA TFNC membrane. However, for the TFC and 0.025-B-GQD/PA TFNC membranes, the water flux in the experimental study was higher. For the 0.050-B-GQD/PA TFNC membrane, the simulated water flux was almost 3750 L/m^2^ h. The trend of simulated data was the same as the experimental data in terms of the membrane with a maximal water flux value, i.e., the 0.05-B-GQD/PA TFNC membrane demonstrated a maximal flux in both the AL-DS (3750 L/m^2^ h) and AL-FS (3810 L/m^2^ h) membrane orientations. With the addition of B-GQDs into the PA layer, the membrane’s water flux increased with 0.025 wt.% of B-GQDs and 0.050 wt.% of B-GQDs, and it subsequently reduced with the 0.075 wt.% B-GQD concentration. Therefore, the presence of nanoparticles in a membrane matrix is a key parameter for increasing water flux [122]. Moreover, Figure 14d,e presents the experimental and simulated solute flux of the TFC and TFNC membranes in the AL-FS and in AL-DS modes, respectively. Additionally, Figure 14f presents the experimental and simulated solute flux percentage deviation in both the AL-FS and AL-DS modes. It was noted that the 0.050-B-GQD/PA TFNC membrane demonstrated a higher solute flux in the simulation study in both the AL-DS and AL-FS modes. Like the solute flux experimental values, there was a decreasing trend in solute flux due to loading of GQD particles. In the simulation study, the 0.05-B-GQD/PA TFNC membrane demonstrated a solute flux reduction of about 93.7 % in the AL-FS orientation and a 89.43% reduction in the AL-DS membrane orientation. Here, the 0.05-B-GQD/PA TFNC membrane in the AL-FS orientation showed a reduced solute flux value of 7.23 g/m^2^ h compared with other membranes developed in this study. Therefore, the water flux and solute flux results were quite acceptable, though there was little difference between the simulation and experimental results.

**Figure 15:**
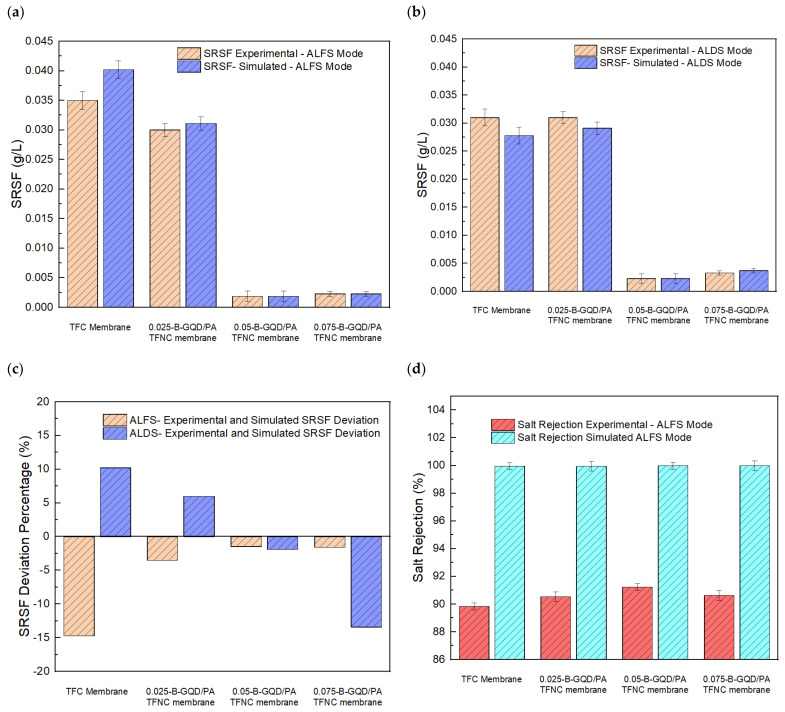

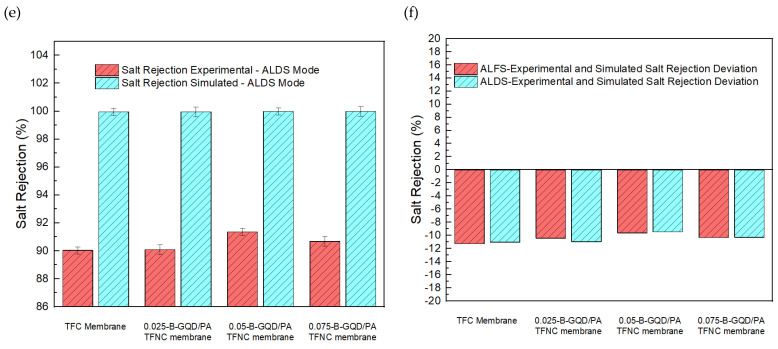
(**a**) AL-FS mode experimental and simulated SRSF; (**b**) AL-DS mode experimental and simulated SRSF; (**c**) AL-FS and AL-DS mode experimental and simulated SRSF percentage deviation; (**d**) AL-FS mode experimental and simulated salt rejection; (**e**) AL-DS mode experimental and simulated salt rejection; (**f**) AL-FS and AL-DS mode experimental and simulated salt rejection percentage deviation.

Additionally, Figure 15a,b shows the experimental and simulated SRSF of the TFC and TFNC membranes in the AL-FS and AL-DS modes, respectively. Additionally, Figure 15c presents the experimental and simulated SRSF percentage deviation in both the AL-FS and AL-DS modes. It was noted that the 0.05-B-GQD/PA TFNC membrane demonstrated the smallest percentage deviation of SRSF, i.e., 1–2%. For the 0.05-B-GQD/PA TFNC membrane, the SRSF value was greater in the simulation study than in the experimental study. In the simulation study, the 0.05-B-GQD/PA TFNC membrane demonstrated reduced SRSF values (0.0023 g/L in the AL-DS membrane orientation and 0.0019 g/L in the AL-FS membrane orientation). With the rise in the B-GQD concentration, it was observed that the SRSF value initially reduced and reached the minimum value with the 0.05 wt.% GQD addition. Consequently, the SRSF value marginally increased with 0.075 wt.% of B-GQDs, which proved the reverse trend of the solute rejection of the TFC membranes. Thus, the simulation results for SRSF data were in good agreement with the experimental study results. Furthermore, Figure 15d,e shows the experimental and simulated salt rejection of the TFC and TFNC membranes in the AL-FS and AL-DS modes, respectively. Moreover, Figure 15f presents the experimental and simulated salt rejection percentage deviation in both the AL-FS and AL-DS modes. Similar to the SRSF modeling results, the 0.05-B-GQD/PA TFNC membrane showed the smallest percentage deviation of salt rejection of the different simulated membranes. Additionally, for the 0.05-B-GQD/PA TFNC membrane, a higher salt rejection was noted in the simulation study compared with the experimental study. Overall, it can be noted that the highest water flux, the lowest solute flux, the minimal SRSF, and the maximum salt rejection were noted in the simulation study for the 0.05-B-GQD/PA TFNC membrane, which was the same as that observed in the experimental study in both the AL-FS and AL-DS modes. Thus, the simulation results were in agreement with the experimental results.

## 4. Conclusions

In the current study, a new type of TFC membrane was developed by incorporating green-synthesized GQDs into the PA layer and using a PES nanofiber membrane prepared using the SBS method as the intermediate support layer. GQDs from banyan tree leaves were prepared for the first time using a facile and green single-pot hydrothermal method using just DI water, which involves no extra reducing agent or organic solvent. The B-GQDs were characterized by the UV–vis, PL, TEM, Raman, and FTIR analysis to confirm their successful preparation. The TEM results established that the synthesized B-GQDs had particle sizes ranging from 2 to 5 nm. The GQD-incorporated TFC membranes demonstrated improved surface hydrophilicity (34%) and smoothness (17.3%) compared with the pristine thin-film composite membrane. An analysis of impact of B-GQD concentration on the membrane performance in the FO process, a long-term performance assessment of the TFNC membrane, and a chlorine resistance examination of the TFC and TFNC membranes were performed. It was observed that the forward osmosis performance of the thin-film composite membranes was enhanced with the inclusion of 0.050 wt.% of B-GQDs into these membranes. All membranes demonstrated an increased water flux in the AL-DS membrane orientation compared with than in the AL-FS membrane orientation. The 0.050-B-GQD/PA TFNC membrane demonstrated an improved water flux, reduced SRSF, decreased solute flux, and better salt rejection than the TFC membrane. Moreover, in the simulation study, it was noted that the highest water flux, the lowest solute flux, the minimal SRSF, and the maximum salt rejection were noted for the 0.05-B-GQD/PA TFNC membrane, same as that observed in the experimental study in both the AL-FS and AL-DS modes. Thus, the simulation results were in agreement with the experimental results. The current study establishes that banyan tree leaf-derived B-GQD-incorporated thin-film composite membranes with a SBS NFM substrate show an enhanced FO performance regarding water flux, salt rejection, and chlorine resistance ability. Therefore, the membranes developed in the current study have great prospective applications in wastewater treatment, water purification, and desalination.

## Figures and Tables

**Table 1:** Water content analysis of TFC and TFNC membranes.

	Water Content of the Membrane (%)
Nanofiber Membrane	62.00
TFC membrane	64.25
0.025-B-GQD/PA TFNC membrane	65.09
0.050-B-GQD/PA TFNC membrane	67.03
0.075-B-GQD/PA TFNC membrane	70.23

**Table 2:** Membrane-intrinsic property evaluation results.

	Water Permeability (A) in LMHB	Salt Rejection Obtained in RO System in % (R)	Salt Membrane Permeability (B) in LMH	Structural Parameter (S) in m
TFC membrane	44.43	89.01	1.51	1 × 10^−6^
0.025-B-GQD/PA TFNC membrane	46.11	90.65	1.43	2 × 10^−6^
0.050-B-GQD/PA TFNC membrane	50.16	91.89	0.09	2 × 10^−6^
0.075-B-GQD/PA TFNC membrane	48.64	90.45	0.11	4 × 10^−6^
